# Real Time Pear Fruit Detection and Counting Using YOLOv4 Models and Deep SORT

**DOI:** 10.3390/s21144803

**Published:** 2021-07-14

**Authors:** Addie Ira Borja Parico, Tofael Ahamed

**Affiliations:** 1 Graduate School of Life and Environmental Sciences, University of Tsukuba, Tennodai 1-1-1, Tsukuba, Ibaraki 305-8577, Japan; parico.addie.rw@alumni.tsukuba.ac.jp; 2 Faculty of Life and Environmental Sciences, University of Tsukuba, Tennodai 1-1-1, Tsukuba, Ibaraki 305-8577, Japan

**Keywords:** YOLO, YOLOv4, Deep SORT, object counting, real time, object detection, fruit detection

## Abstract

This study aimed to produce a robust real-time pear fruit counter for mobile applications using only RGB data, the variants of the state-of-the-art object detection model YOLOv4, and the multiple object-tracking algorithm Deep SORT. This study also provided a systematic and pragmatic methodology for choosing the most suitable model for a desired application in agricultural sciences. In terms of accuracy, YOLOv4-CSP was observed as the optimal model, with an AP@0.50 of 98%. In terms of speed and computational cost, YOLOv4-tiny was found to be the ideal model, with a speed of more than 50 FPS and FLOPS of 6.8–14.5. If considering the balance in terms of accuracy, speed and computational cost, YOLOv4 was found to be most suitable and had the highest accuracy metrics while satisfying a real time speed of greater than or equal to 24 FPS. Between the two methods of counting with Deep SORT, the unique ID method was found to be more reliable, with an F1count of 87.85%. This was because YOLOv4 had a very low false negative in detecting pear fruits. The ROI line is more reliable because of its more restrictive nature, but due to flickering in detection it was not able to count some pears despite their being detected.

## 1. Introduction

Pear farmers in Japan typically count their yield manually and tend to have higher post harvest losses due to short perishability and packaging arrangements. In addition, quicker decision making is necessary in an extreme climatic event situation. Japan is vulnerable to typhoon disasters, which may lead to huge losses in pear orchards. To make this process easier for farmers, a mobile phone-based application for counting pears in real-time was conceptualized to support the logistics management of pears. However, this would require a fast and accurate detection method that is not computationally expensive. Taking a video from under the trees would require robustness due to challenges such as illumination and occlusion.

Deep learning algorithms have proven to be the most robust way for object detection [1,2]. Considering accuracy and speed, YOLOv4 (You Only Look Once) [3] has been the top performer for object detection models recently. YOLOv4 runs two times faster than a recent state-of-the-art object detection model, EfficientDet, at a similar accuracy. More importantly, YOLOv4 was designed to enable training on a single conventional GPU, unlike other models. After the development of YOLOv4, Wang et al. [4] modified the structure of YOLOv4 to enable scaling for different applications. YOLOv4-tiny was designed to maximize speed and to achieve the minimum computational cost possible. Then, YOLOv4-CSP and other larger versions of YOLOv4 were developed to maximize accuracy with varying computational requirements. In this study, the authors evaluated the speed-accuracy-memory tradeoff among YOLOv4, YOLOv4-tiny and YOLOv4-CSP on detecting pear fruits.

One can use detection alone for counting objects. However, some of the most common problems in detection systems are flickering, failure to detect the object under occlusion and challenging illumination. Therefore, relying completely on the number of detections for the pear count could lead to errors, especially in a pear orchard scenario where the abovementioned challenges are common. For that reason, a back-up system should cover for this limitation to ensure accuracy in counting, which can be through object tracking. With object tracking, a unique ID can be assigned to a detection, thus, giving a more reliable measure of object count just in case the detection system fails. Deep SORT (Simple Online Realtime Tracking with Deep Association Metric) has been proven to be one of the fastest and most robust algorithms for multiple object tracking (Wojke et al. 2017). Deep SORT was found to have runtime speed of 25–50 FPS using recent conventional GPUs [5]. Due to its suitability for real-time tracking and robustness, Deep SORT was selected as the tracking algorithm in this study for counting the pear fruits in real time.

Several studies have utilized YOLO-based models for fruit detection and have demonstrated that YOLO models have a huge potential in accurate real time detection of fruits in an orchard [6,7,8,9,10,11,12,13,14,15,16]. However, there were some concerns found among these studies. First, no study simultaneously considered the parameters of detection accuracy, inference speed and computational cost, which are very important for the optimization of the detection algorithm. Second, most of the studies did not report loss curves for their models, thus, so it is difficult to verify if overfitting or underfitting of their dataset occurred. It is also difficult to confirm if they had truly achieved the highest possible performance metrics exhaustively without overfitting. Finally, reported research only focused on detection and did not address the possibility of counting the objects in real time, which is a completely different matter.

There was one study found where YOLO was combined with a classical tracking algorithm (Kalman filter) for counting the fruits with an F1 score of 0.972 [17]. However, the speed of the counting system was not mentioned. In addition, it was not stated if the information processing was done in one batch or sequentially. Thus, it is difficult to confirm if their system is truly applicable in real time.

Considering these limitations in related studies, the main contribution of this study was to develop a real time fruit counting system through detection and tracking while evaluating the computational cost. The concept is that the pear counter counts the pear fruits from a video taken in real time based on a two-part system with a state-of-the-art detection algorithm YOLOv4, and a leading tracking algorithm Deep SORT. To do that, detection performance, inference speed and computational cost were considered concurrently as target metrics for optimizing the detection algorithm.

Furthermore, a comprehensive, systematic, and pragmatic guide in performing an object detection study in an agricultural or life sciences application was provided. First, target metrics were clearly defined. Then, a modified data splitting scheme was proposed for cases where data mismatch existed between the available training and test datasets. Next, this research also provided a methodological training strategy that can guide the researcher to objectively diagnose problems that exist during training, such as overfitting, and underfitting. With the systematic guide provided in carrying out object detection research, high quality and reproducible results are ensured.

This article provides the problem statement and contributions to the agricultural production system for minimizing post harvest losses in Section 1. In Section 2, more details about the YOLO models, Deep SORT, and other related studies are discussed. Section 3 lays out the systematic methodology in preparing the data, defining the target metrics, carrying out the training, validation, and optimization of the detection models, comparison of the YOLOv4 models (YOLOv4, YOLOv4-CSP, and YOLOv4-tiny), integration with the tracking algorithm and, finally, evaluation of the pear counting system. In Section 4, the results of the training, error analysis, model comparison and evaluation of the pear counting system are presented. Finally, Section 5 outlines the conclusion and future directions of the study.

## 2. Related Works

### 2.1. You Only Look Once (YOLO)

**Figure 1:**
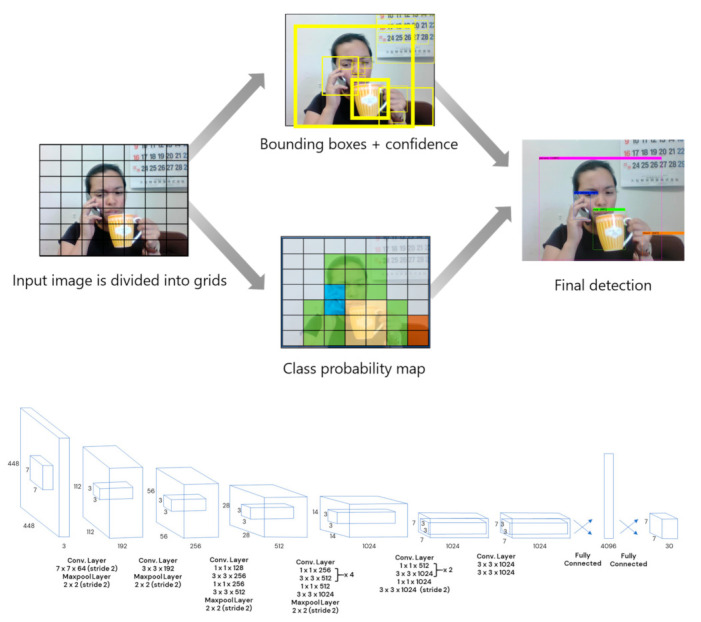
(**Top**) General workflow of YOLO (You Only Look Once); (**Bottom**) YOLO Architecture.

Deep learning algorithms have been shown to be one of the most robust ways for approaching object detection [1]. Considering accuracy and speed, YOLOv4 [4] has been the top performer for object detection models recently. Joseph Redmon in 2016 developed the predecessor of YOLOv4, You Only Look Once, also known as YOLO, which was considered one of the first CNNs with real-time speed. Its speed was attributed to its one-shot detection mechanism, where it simultaneously predicted the bounding box coordinates and class probabilities from an image [18]; thus the term “You Only Look Once”. YOLO divides an input image into S x S grids then predicts bounding boxes with corresponding confidences of having detected an object of class C (Figure 1). A threshold value is set to eliminate bounding boxes of low confidence. Therefore, probabilities that are greater than the threshold value are considered detections.

However, the YOLO had its downsides. It had difficulties in detecting small objects and objects with unusual aspect ratios. It also made more localization errors compared to the state-of-the-art object detection model, Fast R-CNN. In 2017, a more accurate counterpart of YOLO was introduced, which was called YOLOv2. The accuracy improvements in YOLOv2 were mainly due to the use of anchor boxes in predicting the location of objects in an image, batch normalization in the convolutional layers, and high-resolution classifier. Because of these improvements, YOLOv2 was able to outperform some state-of-the-art models such as Faster R-CNN with a mAP of 78.6% and an inference speed of 40FPS on a Pascal VOC 2007 dataset and an mAP0.5:0.95 of 21.6% on an MS COCO test-dev 2015 [19].

Then, a year after, several improvements were applied to YOLOv3. In this version, the previous backbone network of YOLOv2 (Darknet-19) was replaced with Darknet-53. Other than that, the following were also integrated into the system: (1) binary cross entropy in loss calculations, (2) use of logistic regression in predicting the “objectness score” for each bounding box and (3) feature extraction at three different scales inspired by FPN [20,21]. Because of these enhancements, compared to YOLOv2, YOLOv3 had a better AP of 28.2% on an MS COCO dataset, which was on par with SSD but three time faster. However, the increased accuracy had some cost on the inference speed.

In early 2020, Bochkovskiy et al. [3] introduced YOLOv4, which is more accurate and faster than YOLOv3 by 10% and 12%, respectively. YOLOv4 runs two times faster than a recent state-of-the-art object detection model, EfficientDet, at a similar accuracy. More importantly, YOLOv4 was designed to enable training on a single conventional GPU, unlike other models. The efficiency and increase in accuracy of YOLOv4 in object detection result mainly from several improvements incorporated into the model, which are: (1) cross-stage partial connections (CSP) in the new CSPDarknet53 inspired by CSPNet [22], (2) use of Mish and Leaky ReLU as an activation function [23,24], (3) adoption of a Path Aggregation Network [25] in place of the FPN that was used in YOLOv3 and (4) use of Spatial Pyramid Pooling [26] as a plug-in module.

After the development of YOLOv4, Wang et al. [4] modified the structure of YOLOv4 to enable scaling for different applications. YOLOv4-tiny was designed to maximize speed and to achieve the minimum computational cost possible. Then, YOLOv4-CSP and other larger versions of YOLOv4, were developed to maximize accuracy with varying computational requirements. In this study, the author compared the performances of YOLOv4, YOLOv4-tiny and YOLOv4-CSP on detecting pear fruits.

One can use detection alone for counting objects. However, some common problems for detection systems are flickering and failure to detect the object under occlusion and challenging illumination. Therefore, relying completely on the number of detections for the pear count would be erroneous, especially in a pear orchard scenario where the mentioned challenges are common. For that reason, a back-up system should cover for this limitation to ensure accuracy in counting, which can be through object tracking. With object tracking, a unique ID can be assigned to a detection, thus, giving a more reliable measure of object count in case the detection system fails.

### 2.2. Simple Online and Real Time Tracking with Convolutional Neural Networks

Among the algorithms for multiple object tracking, Deep SORT has proven to be one of the fastest and most robust approaches [5]. It started as the Simple Online and Real time Tracking (SORT) algorithm [27], which was developed to have a minimalistic approach in detection-based online tracking, which focused on efficiently associating object detections on each frame. It took advantage of the high reputation of convolutional neural networks in accurately detecting objects. In addition, two classic methods in motion prediction and data association, the Hungarian algorithm [28] and Kalman filter [29], were implemented as the tracking components. Due to its modest complexity, SORT was 20 times faster than other state-of-the-art trackers [27]. Using Faster R-CNN [30] as the detector, it also had better performance compared to the traditional online tracking methods in the MOT (Multiple Object Tracking) Challenge 2015 [31].

**Figure 2:**
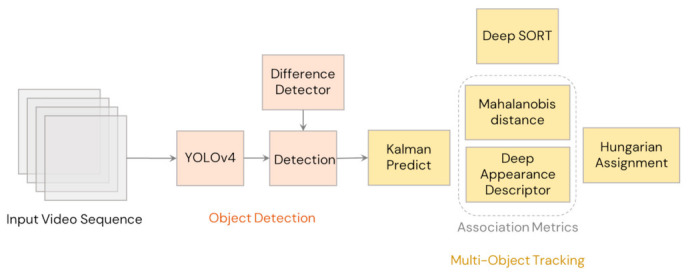
Architecture of Deep SORT (Simple online and real time tracking with deep association metric).

The main drawback with SORT was occlusions and when viewpoints change. To solve this issue, Wojke et al. [5] developed Deep SORT, which is an extended version of SORT (illustrated in Figure 2). In Deep SORT, instead of relying only on motion-based metrics in data association, it also integrated a deep appearance-based metric derived from the convolutional neural network. This change resulted higher robustness from occlusion, change in viewpoint, and in using a nonstationary camera for lower identity switches. Using a modern GPU, Deep SORT was found to have a runtime speed of 25–50 FPS using recent conventional GPUs [5]. Due to its suitability for real-time tracking and robustness, Deep SORT was selected as the tracking algorithm in this study for counting the pear fruits in real time.

### 2.3. Fruit Detection Using YOLO

Several studies have utilized YOLO-based models for fruit detection. Koirala et al. [6] performed real time mango fruit detection with their Mango-YOLO model that exhibited an F1 score of 0.968, AP of 98.3% and an inference speed of 14 FPS on a NVIDIA GeForce GTX 1070 Ti GPU. Liu et al. [7] and Lawal [9] also proposed their own YOLOv3-based tomato detection systems that showed similarly remarkable AP values of 96.4% and 99.5%, respectively, and a faster speed of around 19 FPS for both studies using an NVIDIA GeForce GTX 1070 Ti GPU and an NVIDIA Quadro M4000 GPU, respectively, but this was still not sufficient for real time detection (≥24 FPS). Li et al. [10] also developed Lemon-YOLO for detecting lemon fruits, where they replaced Darknet-53 with an SE_ResGNet34 network. Their system had an AP of 96.28 % and a detection speed of 106 FPS on the high-powered Tesla V100 GPU. Other studies also evaluated the performance of YOLO-based models on detecting other fruits such as apple, lemon, banana and cherry [8,11,12,13,15,16,32].

It is important to note that most of the studies did not report loss curves for their models, thus, it is difficult to verify if overfitting or underfitting of their dataset occurred. It is also difficult to confirm if they truly achieved the highest possible performance metrics exhaustively without overfitting.

### 2.4. Real Time Fruit Counting Using YOLO and an Object Tracking Algorithm

Only one study combined YOLO with a multiple object tracking algorithm for counting fruits. Itakura et al. [17] used YOLOv2 and Kalman filter to count pear fruits from a video to achieve an AP of 97% in detection and an F1 score in counting of 0.972. However, the speed of their counting system was not mentioned. It was also not stated if the tracking algorithm approach was online (current predictions rely only on past information) or offline (processes all information in one batch). Online tracking is more suitable for real time counting. Although offline tracking (also called batch tracking) can be more accurate, processing only occurs after all observations are obtained, thus making it an unattractive option for real time counting. Because of the lack of information about their tracking approach, it is difficult to confirm if their system is truly applicable in real time.

## 3. Materials and Methods

### 3.1. Field Data Collection

**Figure 3:**
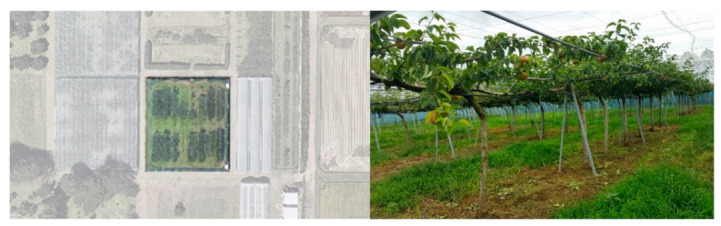
Aerial view of the joint-tree pear orchard (**left**). Joint-tree pear orchard in the Tsukuba-Plant Innovation Research Center, University of Tsukuba, Tsukuba, Ibaraki (**right**).

RGB video acquisition was done using two different cameras: a DJI Osmo Pocket, with a 12MP, 1/2.3″ CMOS sensor and field of view of 80° F2.0, and a mobile phone camera (16 MP, 1/2.6″ BSI CMOS Sensor, F1.9 lens, with OIS). Video acquisition was done in a 0.15 ha joint-tree pear orchard (as seen in Figure 3) in the Tsukuba-Plant Innovation Research Center, University of Tsukuba, Tsukuba, Ibaraki (36°06′56.8″ N, 140°05′37.7″ E) on a cloudy day and a partly cloudy day. The videos were taken from the bottom side of the trees. The details regarding the videos are outlined in Table 1.

### 3.2. Data Preparation

#### 3.2.1. Videos Were Converted into Image Frames

Videos were converted into image frames using the “Scene video filter” of VLC. An automatic screenshot of the video was taken at an interval of half a second. For example, if the video had a frame rate of 60 FPS, one image frame was taken every after 30 frames. For a 30 FPS video, one image frame was taken every 15 frames.

Next was the elimination of images without pear fruits from the dataset. After that, the remaining image frames were 314 4k resolution images and 134 1920 × 1088 resolution images, giving a total of 448 images. The dataset was further expanded through augmentation.

#### 3.2.2. Labelling

Bounding box labelling was done using Supervisely^®^. Its labeling interface allows precise and efficient labelling, reducing the possibility of human-error in labelling ground truth data. However, the export format of the labels was only in Supervisely format (JSON). Other opensource Python-based annotation tools that export directly in YOLO format include LabelImg [33] and OpenLabeling [34].

Roboflow^®^ was used to convert the Supervisely format labels to the desired frameworks (such as Darknet, Tensorflow™, PyTorch, etc.). An alternative to Roboflow is an open-source method of converting Supervisely labels to YOLO format [35].

#### 3.2.3. Data Augmentation

It is essential to make the pear fruit detection system more robust to different scenarios through diverse representation of pear fruits in joint-tree systems in one’s dataset. However, there may be unavoidable bias present in the dataset that may not be obvious to the researcher, which can cause overfitting on the training dataset. To mitigate this possible concern, there is an assumption that more information can be extracted from the training dataset if the images are transformed in different ways. This is called data augmentation, which may simulate a wider representation of pear fruit data in orchards, thus avoiding possible overfitting to the training dataset. However, how does one decide which data augmentation techniques should be used?

There are two types of data augmentation: pixel-level and spatial-level. Pixel-level transformations change the images themselves but leave the bounding boxes unchanged. Some examples of pixel-level transformations are blurring, changing the brightness or exposure, adding noise, Cutout, Cutmix and so on. This is useful if the researcher wants to preserve the bounding boxes themselves and intends to not distort the shape of the target object. Spatial-level transformations, on the other hand, change both the image and the bounding box, which makes the transformation slightly more complicated to code compared to pixel-level transformations. However, spatial-level transformations were shown to be more effective in improving the performance of object detection systems [36]. In this study, both kinds were used.

The following image transformations were done to the 4k images.

- Random flip (horizontal or vertical.)
- Random brightness adjustment from −25% to +25%.
- Random adjustment of gamma exposure from −20% to +20%.
- Coarse Dropout: up to 6% of the image’s pixels were subject to noise.

Images were resized to the following sizes: 416 × 416, 512 × 512 and 608 × 608. The aspect ratios of the images were preserved by adding black padding to avoid distorting the aspect ratio of the pear fruits. Through augmentation, the dataset was expanded from 448 images to 1337 images.

### 3.3. Data Splitting

As mentioned in the previous section, the dataset was comprised of 1337 images. We adapted a rule-of-thumb in data splitting for dataset sizes from 100 to 10,000, which was 70:30 for training and validation sets. This is typical for datasets that have even distribution, meaning the training and validation set are not too different from each other. However, in this study, the dataset had uneven distribution. High-resolution images were used in training the neural networks to enable them to detect smaller objects. Then, the trained neural networks were tested on the target application of this study, which were lower resolution mobile phone images. One may think that validating the trained model with a dataset that has a different distribution does not truly evaluate the performance of the trained model. However, how can the “learning” performance be truly measured?

Considering the uneven distribution, the dataset was split into four parts in a 70:10:10:10 ratio: training, training-validation, validation and test set. The training and training-validation set contained the high-resolution images. The training-validation was the unseen high-resolution images, and was used to check if the trained model had overfit the training images. Again, the main target of the detection was pear counting using mobile phones. Thus, the validation and test sets were comprised of mobile phone images. The validation set’s purpose was to check if there was a huge data mismatch between the Osmo images and the mobile phone images. The test set, on the other hand, was used to determine if the model had overfit the validation set.

### 3.4. Setting the Target Metric

Before training a network, it is important to set the desired error rate or accuracy. The whole point of training, validation and optimization is to achieve the desired error rate or accuracy in detection. The desired error rate can be set at the same level as the human-level error in application. In pear fruit counting, the error rate was close to zero.

Accuracy is important in counting fruits. However, a machine that is adept in counting would be deemed useless if the speed was not real time. Moreover, for mobile phone use, it is important to consider if the inference should be done using the phone’s local computational resources or through cloud computing. For example, if the inference is desired to be done on the device itself, the pear fruit counter should not require too much computational power. So, for a pear fruit counter using mobile phones, the aim is to have maximum accuracy that satisfies the minimum speed requirement, while considering the GPU consumption.

Thus, the goal was the following:

1. Maximize the accuracy metric given the time-constraints, hardware, and dataset size available.
2. Determine the inference speed of the YOLOv4 family and find out which one has inference speed close to real-time (≥24 FPS).
3. Find out the GPU consumption of the YOLOv4 family in terms of FLOPs. The CPU and GPU consumption is proportional to the number of FLOPs used [37].

### 3.5. Evaluation Metrics for the Detection

The performances of the models were evaluated based on the metrics used in the Pascal VOC Challenge [38], which are listed in Table 2. The first metric is Intersection over Union (IoU), which is the proportion of the overlapping area and combined area of the bounding boxes of the prediction and the ground truth object.

True positive, false positive and false negative values are prerequisites of the other performance metrics. A detection is considered a true positive (TP) detection if the IoU is equal to or greater than 0.5. False positive (FP) predictions are those having IoU with values below 0.5. False negative (FN) detections were the ground truth objects that were not detected at all, or those assigned with low confidence in predictions (eliminated by a certain threshold, which was considered 0.25 in this research). After calculating TP, FP and FN, recall, precision, F1 score and average precision can be derived. Recall is the sensitivity of the detection system. This metric is the ratio of true positive detections to total ground truth objects. Precision is the correctness of the predictions, which is the ratio of the true positive detections to all positive detections. Next is the F1 score, which summarizes the overall performance of detection by incorporating both precision and recall. Finally, average precision (AP) is the area under the precision-recall curve interpolated from 11 points of recall and precision at different confidence thresholds. It is similar to the F1 score in the sense that it is one metric that summarizes the accuracy of a model. However, AP considers the confidence level of the predictions. Thus, this metric is more often used as a target metric for evaluating the performance of the models during training, and also for decision making in choosing the best model among the YOLOv4 models.

### 3.6. Components of the YOLOv4 Models

In this study, the authors compared the performances of YOLOv4, YOLOv4-tiny and YOLOv4-CSP on detecting pear fruits. Table 3 shows the differences among these models in terms of their architectural components. In this section, how the elements of these models contribute to their respective characteristics is discussed more in detail.

#### 3.6.1. Cross-Stage Partial (CSP) Connection

**Figure 4:**
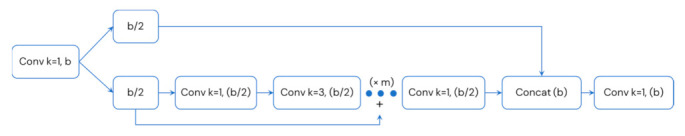
Cross-Stage Partial Connection Block in YOLOv4-CSP.

Cross-stage Partial (CSP) Connection is a technique to reduce computational complexity, which is originally derived from CSPNet [22]. To “CSP-ize” a network divides the feature map of the base layer into two parts then merges the two parts through transition → concatenation → transition (see Figure 4). CSP-ization improves the accuracy and reduces the inference time through truncation of gradient flow [4,22]. Also, CSP-ization enables scaling of the model. Because of these reasons, CSP connections were incorporated into the backbone of the YOLOv4 models. CSPDarknet53 was chosen as the YOLOv4 backbone despite having lower accuracy in image classification compared to CSPResNext50 [3]. The next section explains why.

#### 3.6.2. CSPDarknet53: YOLOv4 and YOLOv4-CSP’s Backbone

Despite CSPResNext50’s better performance in image classification, it was not the case for object detection. CSP-ization of Darknet53 led to higher accuracy in object detection due to the following [4]:

- Higher input network size, which led to the ability to detect more small-sized objects.
- More convolutional layers 3 × 3, which led to a larger receptive field to cover the increased input network size.
- Larger number of parameters for greater capacity to detect multiple objects of different sizes in a single image.

Other than CSP-ization, several techniques were used to improve the performance of CSPDarknet53 without putting a burden on the computational requirement: (1) data augmentation techniques such as CutMix [39] and Mosaic [3], (2) DropBlock [40] as a regularization method and (3) Class label smoothing [3]. Then, the following techniques were used to make the use of expensive GPUs no longer necessary in training: (1) Mish [24] as the activation function (further explained in Section 3.6.4), and (2) Multi-input weighted residual connections [41].

#### 3.6.3. YOLOv4-Tiny’s Backbone: CSPOSANet

**Figure 5:**
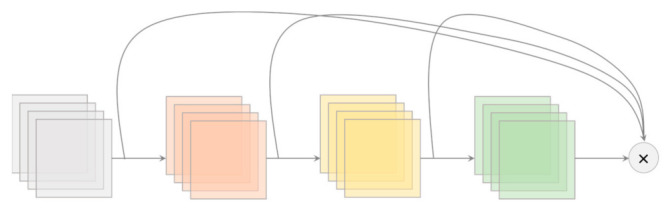
One Shot Aggregation (OSA).

For YOLOv4-tiny, it is important to make the computations efficient and fast without sacrificing much the accuracy. Thus, one shot aggregation (OSA) (shown in Figure 5), which is derived from VoVNet [42], was implemented between the calculation modules of YOLOv4-tiny’s backbone CSPOSANet for smaller computation complexity. This resulted in the reduction of the size of the model and the number of parameters through the removal of an excess amount of duplicate gradient information. A Leaky Rectified Linear Unit was used as the activation function for CSPOSANet due to its faster speed in convergence [23].

#### 3.6.4. Why Were Leaky Rectified Linear Unit and Mish Used as the Activation Functions for the YOLOv4 Models?

**Figure 6:**
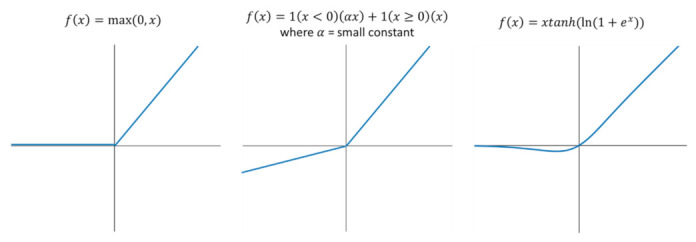
Activation Functions. (**Left**) Rectified Linear Unit (ReLU); (**Center**) Leaky ReLU; (**Right**) Mish.

The Leaky Rectified Linear Unit (or Leaky ReLU) is a modified version of ReLU. The difference is that the former allows a small nonzero gradient over its entire domain, unlike ReLU (Figure 6). Deep neural networks utilizing Leaky ReLU were found to reach convergence slightly faster than those using ReLU. However, Leaky ReLU is slightly less accurate but has lower standard deviations compared to its more novel counterparts Swish and Mish [24]. However, Leaky Re Lu has better performance with under a 75% IoU threshold and with large objects and has lower computational cost due to lower complexity [24].

Mish, on the other hand, is a smooth, continuous, self-regularized, nonmonotonic activation function that enables smoother loss landscapes which helps in easier optimization and better generalization. It has a wider minimum, and thus can achieve lower loss. Because of these benefits, neural networks implementing Mish led to higher accuracy and lower standard deviations in object detection. Moreover, it retains the feature of its predecessors (Swish and Leaky ReLU) in terms of unbounded above and bounded below. The former avoids saturation (which generally causes training to slow down), whereas the latter results in stronger regularization effects (fits the model properly).

Thus, Leaky Re LU would be more suitable if the goal was to maximize speed without sacrificing much of the accuracy. Then, if accuracy should be maximized, Mish would be the better option. Table 4 summarizes the activation functions used and their corresponding effects on each YOLOv4 model.

#### 3.6.5. YOLOv4’s Neck: Path Aggregation Network (PANet)

**Figure 7:**
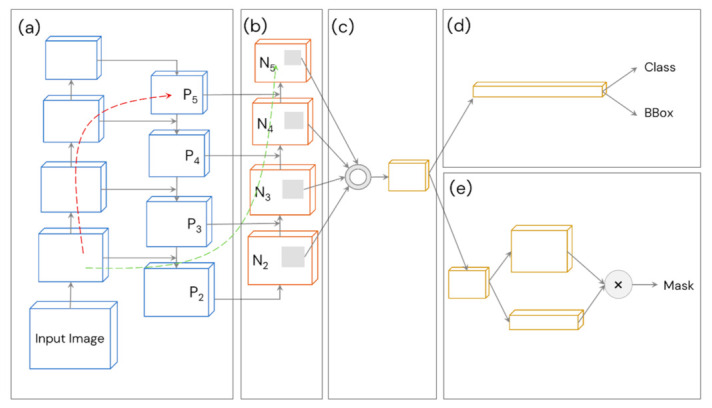
Architecture of PANet, which inspired the path aggregation in YOLOv4’s neck. (**a**) FPN backbone; (**b**) bottom-up path augmentation; (**c**) adaptive feature pooling; (**d**) box branch; (**e**) fully-connected fusion (concatenation is done instead of addition for YOLOv4).

Path aggregation (Figure 7), originally proposed by Liu et al. [25], was used as the neck for YOLOv4 and YOLOv4-CSP in place of FPN (which was used in YOLOv3). This technique aggregates parameters from different backbone levels for different detector levels through bottom-up path augmentation and adaptive feature pooling. Bottom-up path augmentation shortens the information path and enhances the feature pyramid by making fine-grained localized information available to top layers (the classifiers). On the other hand, adaptive feature pooling recovers the broken information path between each proposal and all feature levels (cleaner paths are created). It fuses the information together from different layers using an element-wise max operation. Thus, PANet ensures that important features are not lost. For these reasons, PANet was used as the neck for YOLOv4 and YOLOv4-CSP.

#### 3.6.6. YOLOv4’s Plug-In Module: Spatial Pyramid Pooling (SPP)

**Figure 8:**
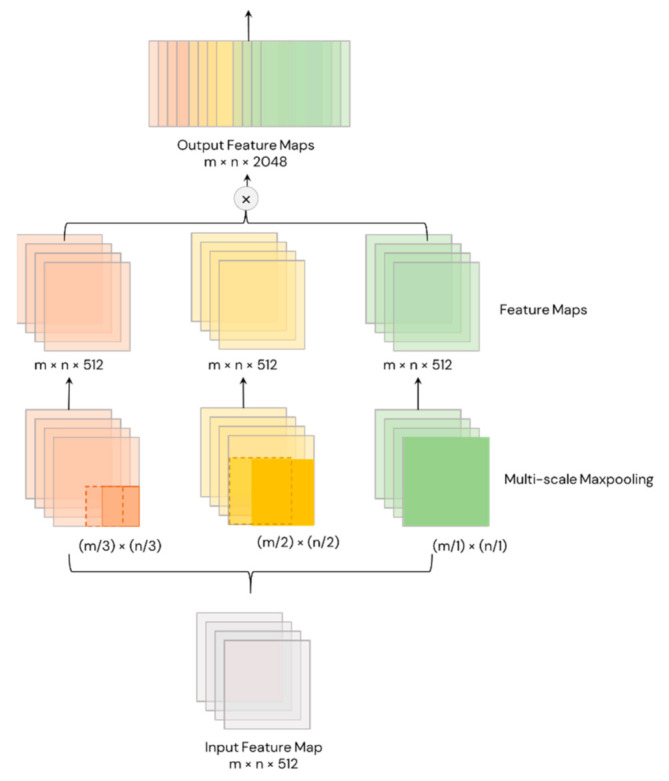
Spatial Pyramid Pooling in YOLOv4.

Spatial Pyramid Pooling (or SPP) is another feature of YOLOv4 and YOLOv4-CSP that eliminates the need for a fixed-size input image, making them more robust and practical. SPP is added on top of the last convolutional layer of YOLOv4 and YOLOv4-CSP. SPP pools the features and generates outputs with fixed-length, which are then fed into the classifier layer (Figure 8). In this study, the pooling was done through the spatially division of feature maps into different scales of d x d equal blocks, where d can be {1, 2, 3, …}. These different scales of division forms are called spatial pyramids. Then, max pooling was done for each level of division to produce a concatenated 1D vector (originally). SPP works similarly in the YOLOv4 models but the difference is the input feature map size is equal to the output feature map size through padding.

### 3.7. Training, Validation and Optimization

The complete training process was composed of two stages: stage-1 training and stage-2 training.

#### 3.7.1. Stage-1 Training

The Darknet framework [43] was used to train the YOLOv4 models within the Google Colab™ Notebook environment. The GPU used for training, validation, and inference was Tesla T4. Custom anchors were calculated using k-means clustering (Table A1 for the custom anchors used). Training-time-augmentation was enabled. The total number of iterations for stage-1 training was 6000.

#### 3.7.2. Hyperparameters

**Figure 9:**
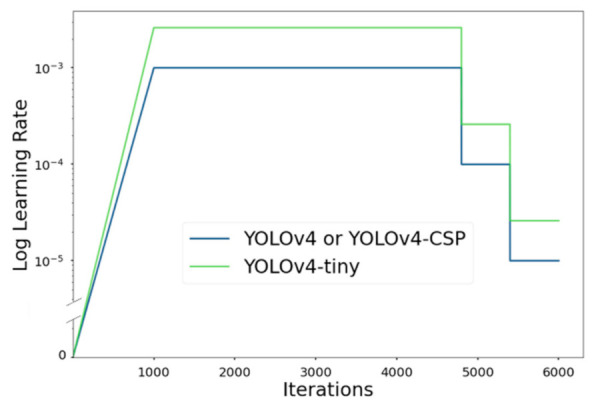
Learning rate schedule of YOLOv4, YOLOv4-CSP, and YOLOv4-tiny, where maximum iteration = 6000. For the first 1000 iterations, a linear warm up policy was done, which is a slow rise of the learning rate. After the 1000th iteration, a multistep decay policy was done.

Stage-1 training used the linear warmup policy for the first 1000 iterations and multi-step decay (other terms: piecewise constant decay, step-wise annealing) as the learning rate schedule policies. The update rule for the multi-step learning rate schedule was as follows:

(1) $$LR_{n+1}=\left\{\begin{array}{c} d\cdot LR_{n},\;if\;n\;in\;\left[steps\right]\\ LR_{n},\;otherwise \end{array}\right.$$

where n is the iteration step, $LR_{n}$ is the previous learning rate, d is the decay rate (d∈ℝ |0<d<1), and $\left[steps\right]$ is the set of iterations when to decrease the learning rate. For this study, d=0.1, $\left[steps\right]=\left[4800,\;5400\right]$, $LR_{0}$= 0.001 for YOLOv4 and YOLOv4-CSP and 0.00261 for YOLOv4-tiny. The learning rate schedules for the YOLOv4 models are illustrated at Figure 9.

**Figure 10:**
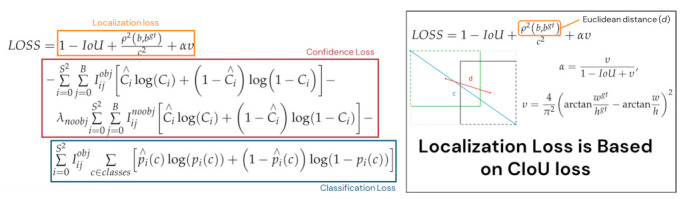
(**Left**) The complete YOLOv4 Loss Function; (**Right**) details of the localization loss based on Complete Intersection-over-Union loss.

Regarding the optimizer, Nesterov Accelerated Gradient, momentum and weight decay were implemented. The momentum and weight decay were set as 0.949 and 0.0005, respectively, for all the YOLOv4 models. The localization loss was based on Complete IoU (CIoU) [44], which is illustrated in Figure 10. A complete list of the hyperparameters is shown in Table A2.

#### 3.7.3. Stage-2 Training

Stage-2 training involved a fine-tuning process, which confirms if the weights with the best mAP from stage-1 training had reached its maximum value. The details of the fine-tuning process can be found in Algorithm A1.

#### 3.7.4. Error Analysis

For a supervised learning algorithm, the best performance achieves low bias and low variance. In typical 70:30 data splitting, the purpose of the error analysis is to determine if the model has achieved the highest possible accuracy without overfitting to the training data.

**Figure 11:**
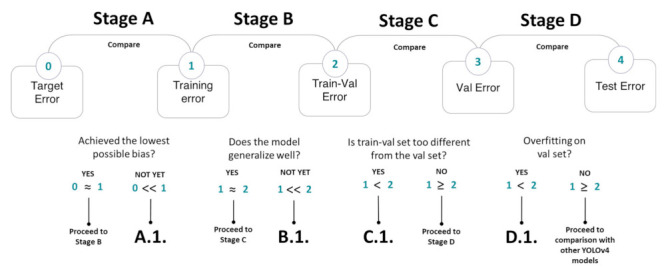
Error analysis to achieve the best possible performance for the model from Stage A to Stage D. The goal of the error analysis is to reduce the gap on each pair of errors by performing the strategies found in Table A4. The target error (also called Bayes error) is the lowest possible detection error, which in this case can be considered as the human error rate in detecting pears.

However, the aim of data splitting at 70:10:10:10 is to train on high resolution images (for more robustness) with the goal of having good performance on low resolution images. Thus, through comparison of each pair of errors in Figure 11, the error analysis answers the following questions:

- Has the model achieved the lowest possible bias?
- Did the model overfit on the training data?
- Does the train-validation set have high mismatch in data distribution compared to the validation set?
- Did the model overfit on the validation set?

To avoid data mismatch at Stage C (Figure 11), data augmentation was done to the training-validation set to simulate a lower image quality, like that of a mobile phone image. After passing all the stages in the error analysis, the optimized models were compared based on their performance on the test set.

### 3.8. Model Comparison

After going through training, validation and optimization, the model that satisfied the following criteria on the test set was chosen to be the YOLOv4 model for the pear counting stage of this study. First, it should be the highest in all evaluation metrics and second, the inference speed should be close to real-time (≥24 FPS). Lastly, the authors noted the GPU consumption of the chosen model to consider if in-device inference or cloud computing inference would be ideal for mobile phone platform implementation.

### 3.9. Pear Counting Using the Selected YOLOv4 Model and Deep SORT

The best performing YOLOv4 model that satisfied the criteria in the model comparison was converted to the Tensorflow™ format. Deep SORT, in combination with YOLOv4, was implemented locally to track the pears in an unseen test mobile phone video of resolution 1080 × 1920, 32 s long, with a frame rate of 30 FPS. The hardware specification was as follows: Quad-core Intel^®^ Core™ i7-7700HQ @2.80GHz, 16.0 GB RAM and NVIDIA GeForce GTX 1060.

**Figure 12:**
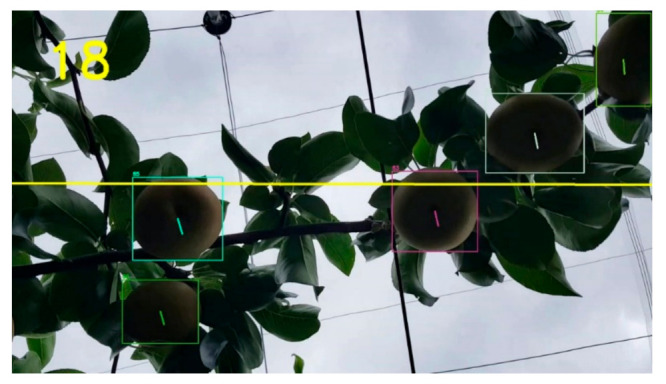
Pear counting system based on YOLOv4-512 and Deep SORT using the ROI-line method. The number on the top left is the number of objects that crossed the horizontal ROI line. Unique IDs can be seen on the top left corner of each bounding box.

Two counting methods were compared in this study: (1) region-of-interest (ROI) method and (2) unique object ID method. The ROI method was based on the number of unique object centroids tracked by Deep SORT that would cross the ROI, which is a horizontal line. Different ROIs were tested, and 50% of the height of the video was deemed to be the optimal ROI. For the second method, the counts were based on the number of unique object IDs generated by Deep SORT’s tracking mechanism. Figure 12 illustrates the pear counting system.

### 3.10. Evaluation Metrics for the Pear Counting

The performance metrics for pear counting are similar to the detection’s evaluation metrics. However, the authors used the subscript *count* to denote metrics associated with pear counting. Additionally, metrics from CLEAR Multiple Object Tracking (MOT) [45], as seen below, were used but modified in the case of this study. The metrics for pear counting are summarized in Table 5. In this study, the objects themselves were not moving, thus, mismatches=0.

## 4. Results and Discussion

The goal of the study was to compare YOLOv4, YOLOv4-tiny, and YOLOv4-CSP in terms of accuracy, speed and memory usage. After evaluating which YOLOv4 model had the best performance in combination with Deep SORT, they were also evaluated for pear counting use.

### 4.1. Training Details

Table 6 outlines the details about the training of the YOLO4 models. YOLOv4-tiny took less than half an hour for 1000 iterations for all the input sizes. This is because smaller models take less time in training due to less computational complexity. In total, it took around 1.4 h to train YOLOv4-tiny completely, which is a remarkably short time. On the other hand, YOLOv4 had a different training speed to that of YOLOv4-CSP despite the similar size. The latter was able to train from 2.0 to 2.8 h for 1000 iterations, compared to YOLOv4, which spanned from 2.0 to 3.1 h. This difference is due to the CSP-ized PAN and SPP architecture of YOLOv4-CSP, which effectively reduced 40% of the computation [4].

**Figure 13:**
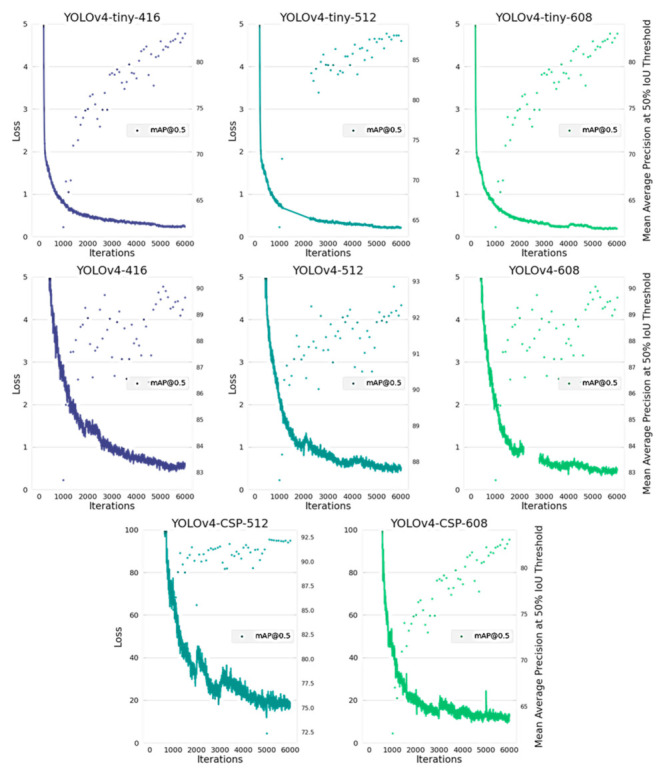
Average Loss and Mean Average Precision (mAP) over 6000 iterations for each of the YOLOv4 models.

However, examining the loss curves in Figure 13, one gets a clue if the models have achieved minimum losses and maximum mAP. Based on the loss graphs, YOLOv4-tiny converged in a quicker manner compared to YOLOv4 and YOLOv4-CSP. This may be due to the fact that it had a higher learning rate and it used the Leaky ReLU as the activation function. Among the models, YOLOv4-CSP seemed to converge the slowest which may be due to the computational cost of CSPization and the use of Mish as the activation function. Upon seeing the differences in convergence rate within 6000 iterations, YOLOv4 and YOLOv4-CSP were further trained following the fine-tuning algorithm from Algorithm A1 to get the best possible weight.

After the fine-tuning process, error analysis was done and the results are shown in Table 7. It can be observed that there is an increasing pattern among the average precision values. Comparing the AP_50_ train-val and AP_50_ val, it can be concluded that the data mismatch between the high-resolution data and lower resolution data was overcome. Looking at the AP_50_ val and AP_50_ test, the values were also increased. This is a good sign that no overfitting occurred on the validation set. Confirming from the error analysis, it is possible to perform comparisons between the performance of the models on the test dataset.

### 4.2. Model Performance Comparison

To reiterate, the authors set the following goals:

1. Maximize the detection performance metrics, which were mentioned at Table 2.
2. Determine which YOLOv4 family has an inference speed close to real-time (≥24 FPS).
3. Find out the GPU consumption of the YOLOv4 models.

YOLOv4, YOLOv4-tiny and YOLOv4-CSP were compared based on the criteria above. Table 8 shows the detection performance of the YOLOv4 models in terms of P, FPR, R, FNR, F1 and Average IoU on the test dataset. YOLOv4-CSP-608 exhibited the best performance among the metrics. This may be due to the Mish used as the activation function and higher network resolution. Thus, the intricate features of the object could be learned at a deeper sense compared to models at lower network resolution.

Interestingly, in terms of P and FPR, most of the models performed well, including YOLOv4-tiny. This shows that even the smallest model had promising accuracy. The second in place in the performance ranking were YOLOv4-512 and YOLOv4-608. An unexpected outcome was that YOLOv4-512 performed better compared to YOLOv4-CSP-512.

**Figure 14:**
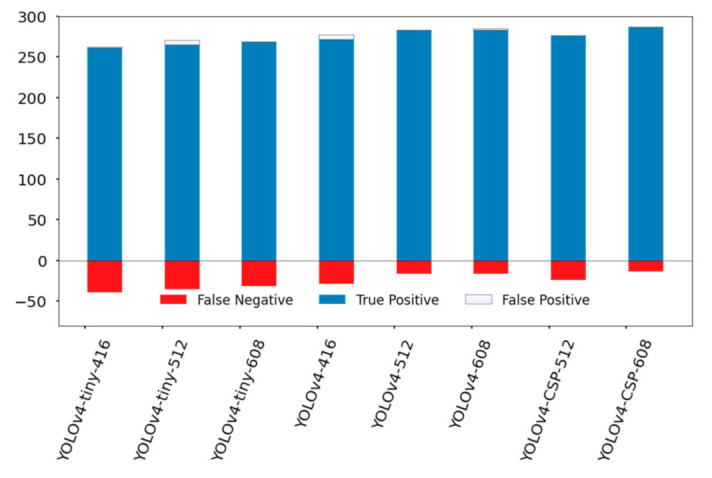
Comparison of YOLOv4 models in terms of the frequency of True Positive, False Positive, and False Negative detections. The specific values are in Table A3.

In counting fruits, FNR was deemed to be better compared to FPR. Looking at Figure 14, consistent with the results from Table 8, the top performing models were YOLOv4-CSP-608, YOLOv4-512 and YOLOv4-608. However, it is difficult to ignore the fact that YOLOv4-tiny-608 had comparable performance to YOLOv4-416 with less false positive detections. This shows the potential of YOLOv4-tiny if a higher network resolution is set. It is possible that YOLOv4-tiny may have satisfactory performance if the network resolution is increased.

### 4.3. Speed-Accuracy Tradeoff in the YOLOv4 Models

**Figure 15:**
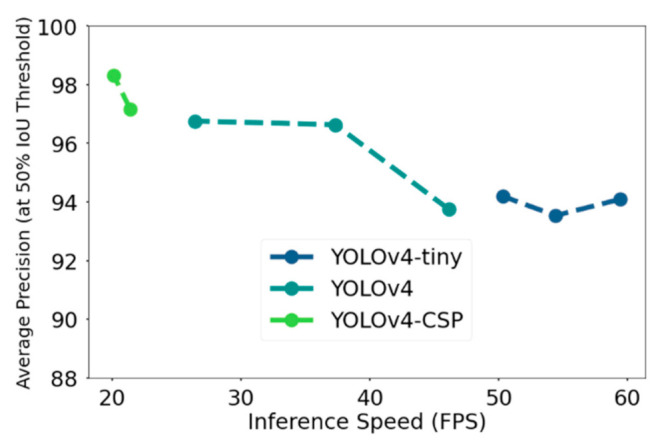
Comparison of the YOLOv4 models in terms of accuracy (in this case, Average Precision at 50% Intersection-over-Union threshold) and inference speed (frame per second).

Figure 15 shows the tradeoff in speed and accuracy in terms of AP_50_ on the test dataset. The specific values can be found in Table A3. YOLOv4-512, 416, and YOLOv4-tiny satisfied the requirement for real-time speed. However, YOLOv4-CSP seemed to have traded off high accuracy with some speed, although it is a good thing to note that the speed of YOLOv4-CSP-512 was very close to real time, which was 21.4 FPS. Another observation was that YOLOv4-608 satisfied the real time speed requirement of ≥24 FPS at 26.4 FPS. Thus, so far, the best model in terms of accuracy while satisfying the real-time speed requirement was YOLOv4-608. The next thing to consider are the metrics in computational power.

### 4.4. Average Precision at Different Thresholds

Average Precision (AP) became more widely used as an accuracy metric because of the PASCAL VOC Challenge and COCO Challenge. AP@0.50 is officially used by the PASCAL VOC Challenge whereas AP@0.75 is considered the “strict metric” for the COCO Challenge. Huang et al. [46] compared different convolutional object detectors in terms of mAP@0.50 and mAP@0.75. Their results showed that their models with low AP at more restrictive IoU thresholds (mAP@0.75) always showed low AP at less restrictive IoU thresholds (mAP@0.50).

**Figure 16:**
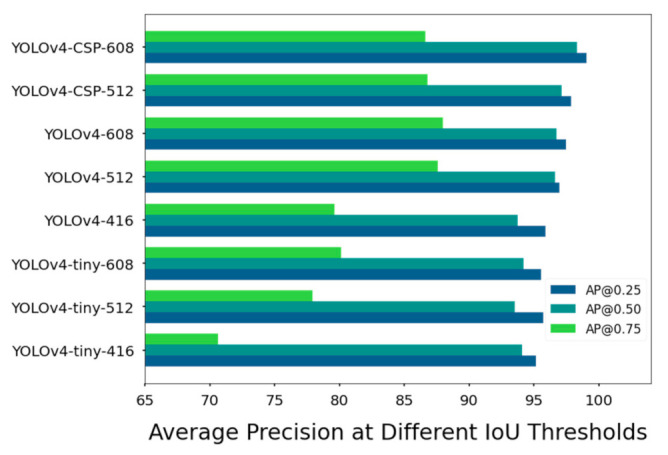
Average Precision (AP) of the YOLOv4 models at 25, 50 and 75% Intersection-over-Union (IoU) thresholds for the test set. The specific values are in Table A3.

However, for the YOLOv4 models, this seemed not to be the case (see Figure 16). A possible reason for the different pattern is the differences in receptive field. Based on observations among state-of-the-art object detection models, the increase in receptive field was found to be associated with higher classification accuracy [47]. Increase in the receptive field is a natural consequence of a higher number of layers, so this is one possible reason why YOLOv4 and YOLOv4-CSP had higher AP.

Nevertheless, it is important to note that receptive field size is not the only contributing factor to the differences in the AP values of the YOLOv4 models. Another factor that may have affected the performance could be the presence of residual connections. As seen in Figure 16, YOLOv4-tiny-608 performed better, if not on par, with YOLOv4-416. Other than an increased receptive field caused by the higher network resolution, the use of a one-shot aggregation technique in pooling data in YOLOv4-tiny and CSP connections could have contributed hugely to its competitive performance. Thus, if YOLOv4-tiny was trained at network resolutions higher than 608 × 608, it might have very satisfactory accuracy metrics while maintaining low computational cost.

### 4.5. FLOPS Analysis

**Figure 17:**
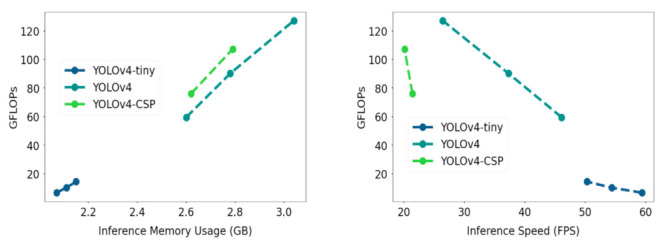
Relationship of Floating Point Operations per Second (FLOPs) with Inference GPU Memory Usage (**left**) and Inference Speed (**right**) for each YOLOv4 model. The specific values are given in Table A3.

Other than metrics measuring the correctness and sensitivity of detection, another important thing to consider is the computational cost of the YOLOV4 models. The inference GPU memory usage for each model was noted and can be seen in Figure 17. To also take into account a platform-independent measure of computation, FLOPS (floating point operations per second) was plotted against the inference GPU memory usage data. As confirmed from the FLOPs vs GPU memory usage plot, YOLOv4-tiny had significantly lower computational requirement compared to YOLOv4 and YOLOv4-CSP. The bigger models YOLOv4 and YOLOv4-CSP had comparable computational requirements.

**Figure 18:**
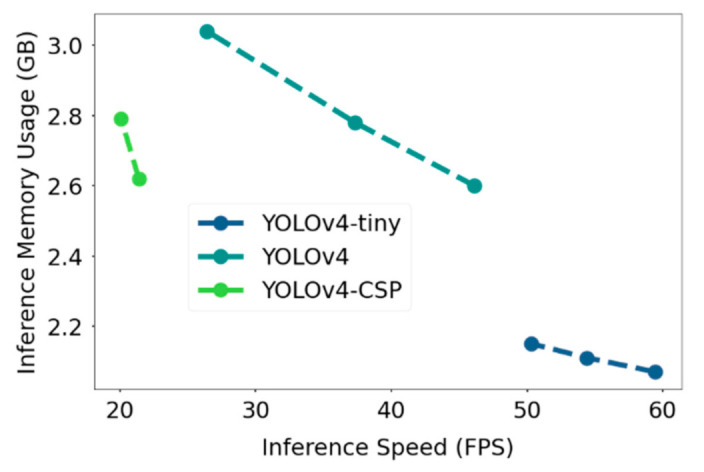
Comparison of YOLOv4 models in terms of speed and GPU memory usage at inference time. The specific values are in Table A3.

In addition, FLOPs was plotted against the inference speed, where the speed-memory tradeoff was observed. YOLOv4-CSP had the slowest inference speed and was observed to have lower FLOPs values at similar network resolutions compared to YOLOv4, which had relatively higher memory consumption but faster speed. YOLOv4-tiny had the best values in terms of speed and computational cost. A similar pattern is seen in Figure 18, where instead of FLOPs, the GPU memory usage was plotted against the inference speed of the YOLOv4 models.

### 4.6. YOLOv4 Models on Illumination and Occlusion Challenges

In an orchard environment, interobject occlusion naturally occurs. Thus, it is important to be able to detect pears despite this challenge. Moreover, in joint tree systems, data acquisition was done from the bottom side of the trees, which caused a high contrast characteristic for the images, making pear detection challenging even for humans.

**Figure 19:**
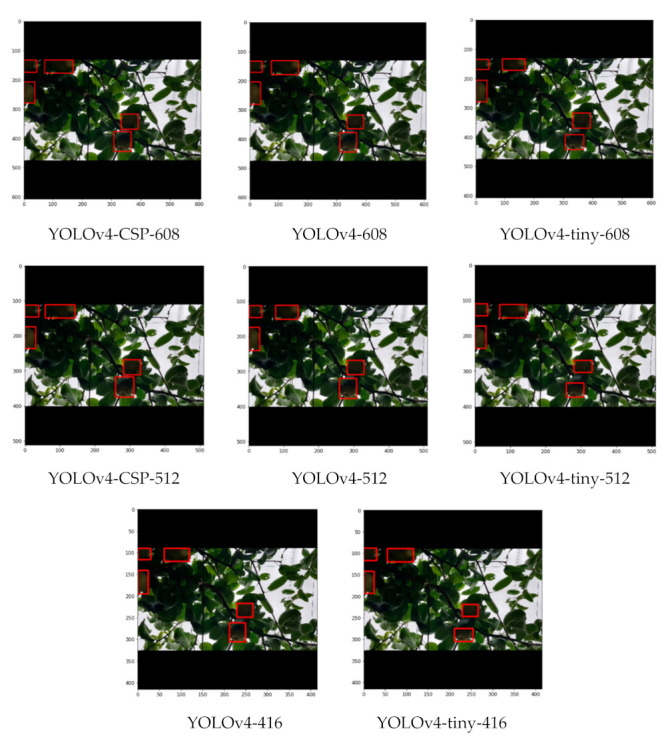
Example detections from the different YOLOv4 models using an image with slight occlusion, potentially-challenging illumination and considerable density of leaves.

**Figure 20:**
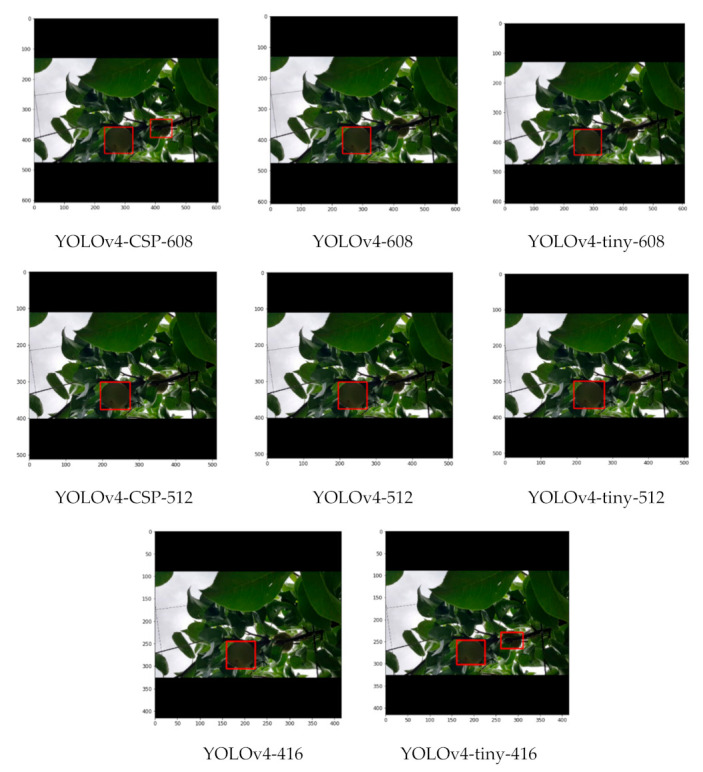
Example detections from the different YOLOv4 models using an image with moderate degree of occlusion and potentially challenging illumination.

**Figure 21:**
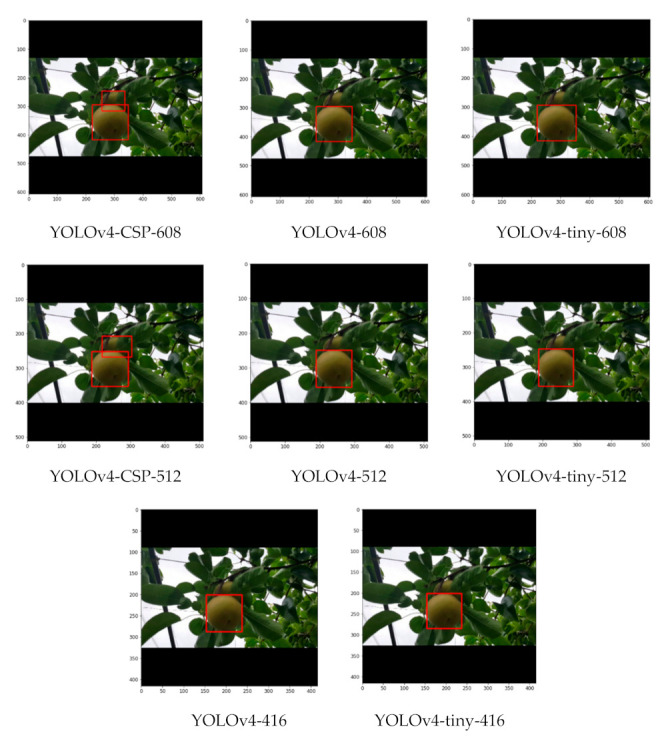
Example detections from the different YOLOv4 models using a close-up image with a high degree of occlusion but good illumination.

**Figure 22:**
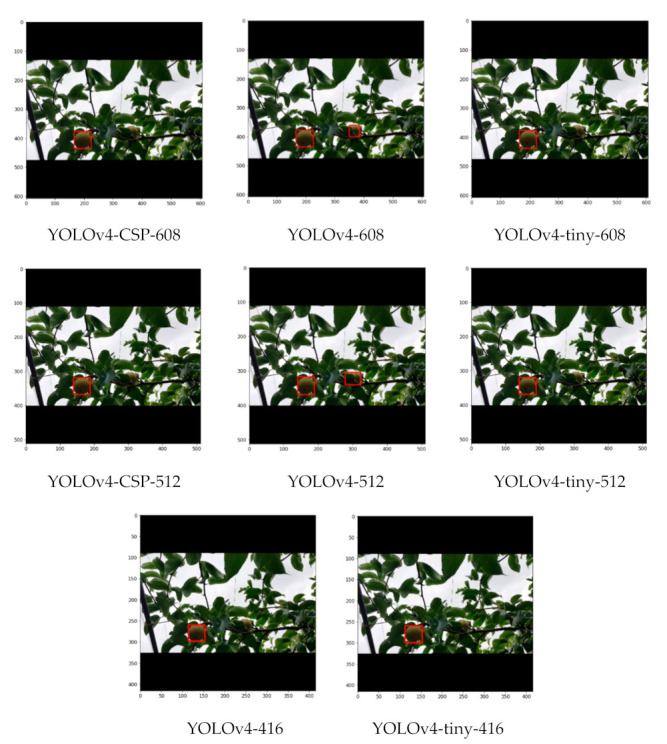
Example detections from the different YOLOv4 models using a nonclose-up image with a moderate degree of occlusion.

As seen from some sample detections in Figure 19, where there is some slight occlusion and potentially challenging illumination, all the YOLOv4 models successfully detected the pears in the image despite having a considerable density of leaves in the background. With higher degrees of occlusion, the differences became more evident. In a still challenging illumination condition, but with a moderate degree of occlusion in Figure 20, YOLOv4-CSP-608 and YOLOv4-tiny-406 (interestingly) successfully detected all the pears. However, in a good illumination condition and with a high degree of occlusion, only the YOLOv4-CSP models were able to successfully detect the pears (Figure 21). YOLO is known to have some difficulty in detecting small objects [48]. From an image with pears that appear smaller and with some degree of occlusion, it was confirmed that YOLOv4-608 and 512 overcame this limitation of YOLO (Figure 22). However, the limitation of YOLO in detecting small pears was exacerbated by the presence of occlusion for the other models.

### 4.7. Comparison of the Pear Counting Methods

Two methods of pear counting were evaluated: through an ROI line and through counting the unique IDs. The differences between the two method’s performances are summarized in Table 9. The unique ID-based method performed better for most performance metrics in counting, specifically on MOTA, FN rate, Recall_count_ and F1_count_. Since the unique ID-based method has a less restrictive nature, it is more sensitive in counting, thus, had lower false negative rate and higher recall. However, the ROI line-based method filtered out false positive detections effectively, as shown by its FP rate of 1.89 percent. Due to its more restrictive nature, it also d more correct detections than the unique ID-based method, as shown by its higher FP rate and Precision_count._ However, the lack of sensitivity of the ROI-line based method was too disadvantageous, resulting in a very low Recall_count_ of 58.49%. Thus, overall, the unique ID-based method had the best sensitivity and correctness tradeoff, as shown by its higher F1_count_ of 87.85%.

### 4.8. Breakdown of the False Negative Counts in the ROI Line-Based Counting

**Figure 23:**
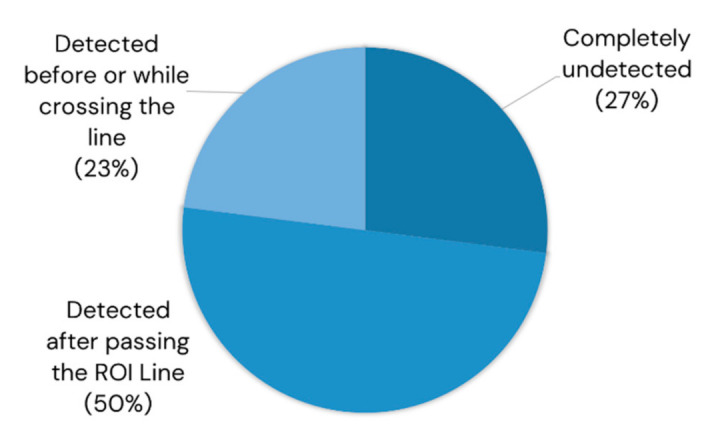
Breakdown of the false negative counts in the region-of-interest line-based counting method.

The ROI line-based method had a low sensitivity in its counts but a very high correctness. How can we improve the sensitivity of an ROI line-based system? The authors observed the behavior of the pear objects that were detected by YOLOv4 but missed by the ROI line, which is summarized in Figure 23. Of the false negative counts, 73% were actually detected by YOLOv4. Of the false negative counts 50% were detected only after passing the ROI Line. This might be due to the limitation of the computational resources, which might have been overcome using a higher-end GPU device. Twenty-three percent of the false negative counts were detected just before or while crossing the line, and this could be attributed to the limitation of Deep SORT’s tracking ability in challenging illumination and increased occlusion due to its reliance on appearance information in tracking. To tackle this limitation, it is recommended to switch the priority to motion information instead of appearance in challenging illuminations to achieve better performance and robustness.

## 5. Conclusions

This study aimed to produce a robust real-time pear fruit counter for mobile applications using only RGB datasets with state-of-the-art object detection models (YOLOv4 models) and the MOT algorithm Deep SORT. In addition, we provided a systematic and pragmatic method for choosing the most suitable model for a desired application in agricultural sciences for further application. In terms of accuracy, YOLOv4-CSP was the optimal model with an AP of 98%. In terms of speed and computational cost, YOLOv4-tiny showed a very promising performance at a comparable rate with YOLOv4 at the lower network resolutions. If considering the balance in terms of accuracy, speed and computational cost, YOLOv4 was found to be the most suitable, with an AP > 96%, inference speed of 37.3 FPS and FN Rate of 6%. Thus, YOLOv4-512 was chosen as the detection model for the pear counting system with Deep SORT. Between the unique ID method and ROI Line method in counting, the former was found to be more reliable compared to the ROI-line method in counting the pears, as characterized by its F1_count_ of 87.85%. It is important to note that this is the case because YOLOv4 had very low false negative detections. The ROI line could be more reliable because of its more restrictive nature, but due to flickering in detection it was not able to count some pears despite their being detected.

To fully maximize the accuracy of detection, cloud computing is recommended with YOLOv4-CSP in mobile applications instead of using the local resources of the mobile phone. If the cost of running a cloud computing service is the concern, Amazon’s cloud services (AWS) support YOLOv4, where 1 million inferences is charged USD 1.362 (2 h) using the server inf1.xlarge in the us-east-1 region. The downside would be requirement to have an internet connection. If on-device inference is preferred, training YOLOv4-tiny at higher network resolutions may be the best option. However, even YOLOv4-tiny requires at least about 2 GB of GPU; thus, low-end mobile phones would not be able to utilize it.

To combat possible flickering problems of the tracking algorithm, counting the unique IDs that tracked for a specific lifespan duration, such as more than 80% of the lifespan, is recommended.

## Figures and Tables

**Table 1:** Details of video acquisition of pear fruits in the joint-tree pear orchard in the Tsukuba-Plant Innovation Research Center, University of Tsukuba, Tsukuba, Ibaraki.

Date	Time	Weather Condition	Device	Resolution, FPS
29 July 2020	12–1 p.m.	Cloudy	Mobile Phone Camera	1920 × 1080, 30
6 August 2020	9–10 a.m.	Intermittently cloudy	DJI Osmo Pocket	3840 × 2160, 60

**Table 2:** Performance metrics that were used to evaluate the YOLOv4 models are described below. This evaluation is based on the Pascal VOC Challenge [38].

Performance Metrics
$Intersection\;over\;Union\;\left(IoU\right)=\frac{area\;of\;overlap}{area\;of\;union}$
$Recall\;\left(R\right)=\frac{TP}{TP+FN}$
$False\;Negative\;Rate\;\left(FNR\right)=1.00-R$
$Precision\;\left(P\right)=\frac{TP}{TP+FP}$
$False\;Positive\;Rate\;\left(FPR\right)=1.00-P$
$F1\;score=\frac{2\cdot P\cdot R}{P+R}$
$Average\;Precision\;\left(AP\right)=\frac{1}{11}\sum_{R_{i}}P\cdot R_{i}$

TP: True Positive, FP: False Positive, FN: False Negative.

**Table 3:** Comparison of YOLOv4, YOLOv4-CSP and YOLOv4-tiny in terms of their architectural elements.

Model Name	Backbone	Neck	Plug-In Module
YOLOv4	CSPDarknet53 + Mish activation	PANet + Leaky activation	SPP
YOLOv4-CSP	CSPDarknet53 + Mish activation	CSPPANet + Mish activation	CSPSPP
YOLOv4-tiny	CSPOSANet + Leaky activation	FPN + Leaky Activation	-

**Table 4:** Summary of activation function used and the reason why the specified activation functions were used.

Model	Activation Function	Effect
YOLOv4	Mish + Leaky ReLU	Balanced accuracy and speed
YOLOv4-CSP	Mish	Maximized accuracy
YOLOv4-tiny	Leaky ReLU	Maximized speed and Minimum computational cost

**Table 5:** The performance metrics used to evaluate the pear counting methods. This evaluation is based on the CLEAR MOT Challenge [45].

Performance Metrics
$R_{count}=\;\frac{TP}{TP+FN}$
$FN\;Rate_{count}=\;1.00-R$
$P_{count}=\;\frac{TP}{TP+FP}$
$FP\;Rate_{count}=\;1.00-P$
$F1_{count}=\;\frac{2\cdot P\cdot R}{P+R}$
$MOTA=\;1-\sum \frac{FN+FP+mismatches}{total\;count}$.

TP: True Positive, FP: False Positive, FN: False Negative, MOTA: Multiple Object Tracking Accuracy.

**Table 6:** Details regarding training YOLOv4 models.

Model Name	Average Training Time per 1000 Iterations (h)	GPU Memory Usage in Training (GB)	Weight Sizes (MB)
YOLOv4-tiny-416	0.22 *	2.22 *	22.96
YOLOv4-tiny-512	0.23 *	2.34 *	22.97
YOLOv4-tiny-608	0.23 *	2.45 *	22.97
YOLOv4-416	2.00	3.08	249.986
YOLOv4-512	2.24	3.18	249.998
YOLOv4-608	3.10	3.34	250.016
YOLOv4-CSP-512	1.91	3.06	205.298
YOLOv4-CSP-608	2.80	4.52	205.316

* subdivisions = 16.

**Table 7:** Average precision for the training-validation set (train-val), validation set (val), and test set (test) IoU threshold = 50% and Confidence Threshold = 0.5.

Model Name	AP_50_ Train-Val	AP_50_ Val	AP_50_ Test
YOLOv4-tiny-416	83.78	92.91	94.09
YOLOv4-tiny-512	86.23	93.08	93.53
YOLOv4-tiny-608	87.61	92.11	94.19
YOLOv4-416	90.39	93.72	93.76
YOLOv4-512	92.86	94.61	96.64
YOLOv4-608	91.32	95.39	96.76
YOLOv4-CSP-512	92.74	93.48	97.16
YOLOv4-CSP-608	92.60	94.51	98.32
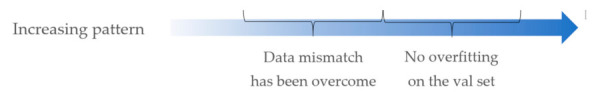			

**Table 8:** Performance metrics of the YOLOv4 models in terms of Precision (P), False Positive Rate (FPR), Recall (R), False Negative Rate (FNR), F1 score and average Intersection-over-Union (IoU) on the test dataset. Ranking is indicated by color, where green is 1st, yellow is 2nd, and orange is 3rd best.

Model Name	P	FPR	R	FNR	F1	Average IoU
YOLOv4-tiny-416	1.00	0.00	0.87	0.13	0.93	83.06
YOLOv4-tiny-512	0.98	0.02	0.88	0.12	0.93	83.13
YOLOv4-tiny-608	1.00	0.00	0.89	0.11	0.94	85.85
YOLOv4-416	0.98	0.02	0.90	0.10	0.94	82.37
YOLOv4-512	1.00	0.00	0.94	0.06	0.97	85.77
YOLOv4-608	1.00	0.00	0.94	0.06	0.97	86.75
YOLOv4-CSP-512	1.00	0.00	0.92	0.08	0.96	86.16
YOLOv4-CSP-608	1.00	0.00	0.95	0.05	0.98	87.18

**Table 9:** Pear counting performance metrics of the pear counting system based on YOLOv4 and Deep SORT on a 1920 × 1080 video between the two approaches in counting: unique-ID based and region-of-interest (ROI) based. Values in bold face are the higher ones between the counting approaches.

Counting Metrics	%	
	Unique-ID Based	ROI-Line Based
MOTA	**75.47**	56.60
FN rate	**11.32**	41.51
FP rate	13.21	**1.89**
Precision_count_	87.04	**96.88**
Recall_count_	**88.68**	58.49
F1_count_	**87.85**	72.94

## Data Availability

The data presented in this study are available on request from the corresponding author.

## Appendix A

**Table A1:** Anchors of the YOLOv4 models.

Model Name	Anchors	Anchor Mask Indices		
		1st	2nd	3rd
YOLOv4-tiny-416	8, 8, 17, 17, 35, 27, 40, 42, 60, 50, 77, 72	5	0, 1, 2, 3, 4	-
YOLOv4-tiny-512	8, 8, 15, 15, 28, 25, 46, 42, 66, 58, 93, 85	4, 5	0, 1, 2, 3	-
YOLOv4-tiny-608	12, 12, 28, 24, 44, 45, 66, 54, 82, 78, 117,100	4, 5	0, 1, 2, 3	-
YOLOv4-416	5, 5, 9, 9, 14, 14, 22, 19, 29, 33, 52, 22, 42, 40, 56, 53, 80, 67	0, 1, 2, 3	4, 5, 6, 7	8
YOLOv4-512	7, 8, 12, 12, 18, 18, 28, 25, 39, 43, 61, 29, 56, 54, 81, 69, 102, 97	0, 1, 2, 3	4, 5, 6	7, 8
YOLOv4-608	8, 9, 15, 15, 26, 22, 37, 34, 24, 60, 61, 43, 63, 64, 85, 78, 117,100	0, 1, 2	3, 4, 5	6, 7, 8
YOLOv4-CSP-512	7, 8, 12,12, 18, 18, 28, 25, 39, 43, 61, 29, 56, 54, 81, 69, 102, 97	0, 1, 2, 3	4, 5, 6	7, 8
YOLOv4-CSP-608	8, 9, 15, 15, 26, 22, 37, 34, 24, 60, 61, 43, 63, 64, 85, 78, 117,100	0, 1, 2	3, 4, 5	6, 7, 8

**Table A2:** Hyperparameters of the YOLOv4 models.

Model Name	Batch	Subdivisions	Momentum	Decay	Initial Learning Rate
YOLOv4-tiny	64	16	0.9	0.0005	0.00261
YOLOv4 or YOLOv4-CSP	64	16	0.949	0.0005	0.001

**Table A3:** Performance metrics of the YOLOv4 models on the Test Dataset (Complete).

Model Name	P	R	F1	Average IoU	AP_25_	AP_50_	AP_75_	BFLOPs	Inference Speed (FPS)	Inference Memory Usage (GB)	TP	FP	FN
YOLOv4-tiny-416	1.00	0.87	0.93	83.06	95.1897	94.0893	70.6543	6.789	59.4	2.07	261	1	39
YOLOv4-tiny-512	0.98	0.88	0.93	83.13	95.7535	93.5346	77.9119	10.283	54.4	2.11	264	6	36
YOLOv4-tiny-608	1.00	0.89	0.94	85.85	95.5740	94.1936	80.1209	14.500	50.3	2.15	268	0	32
YOLOv4-416	0.98	0.90	0.94	82.37	95.9052	93.7559	79.6057	59.571	46.1	2.60	271	6	29
YOLOv4-512	1.00	0.94	0.97	85.77	96.9504	96.6365	87.5749	90.235	37.3	2.78	283	0	17
YOLOv4-608	1.00	0.94	0.97	86.75	97.4674	96.7600	87.9839	127.232	26.4	3.04	283	1	17
YOLOv4-CSP-512	1.00	0.92	0.96	86.16	97.8568	97.1558	86.8072	76.142	21.4	2.62	276	0	24
YOLOv4-CSP-608	1.00	0.95	0.98	87.18	99.0726	98.3243	86.6339	107.359	20.1	2.79	286	0	14

**Table A4:** Strategies in reducing error gap in the error analysis. The stages were defined in Figure 11.

Stage	How to Reduce the Gap?
A.1	Train longer Train a bigger model Use more effective optimization algorithms Momentum, RMS Prop, Adam Try other neural network architectures or hyperparameters
00B.1	Add more to training set Perform regularization methods to improve generalizing ability L2, Dropout, Data augmentationTry other neural network architectures or hyperparameters
C.1	Make training data more similar to val/test set Collect more data similar to val/test set Consider generating synthetic datasetNote: overfitting to the synthetic dataset is a risk
D.1	Increase the val set size

**Algorithm A1**. Pseudocode of the fine-tuning process in Stage 2 training of the YOLOv4 models.
i=0 for 1st training, 1 for 2nd training
$LR_{i}=learning\;rate\;for\;{\left(i+1\right)}^{th}training$
$mAP_{i}=best\;mAP\;for\;{\left(i+1\right)}^{th}training$
N=iteration with the best mAP
$w_{i}=weights\;with\;the\;best\;mAP\;for\;{\left(i+1\right)}^{th}training$
d=decay rate
step1 = first time the learning rate was decreased
if $N_{o}<step1$ then
train at $LR_{1}=d\times LR_{0}$ for $N_{o}+1000$
if $mAP_{0}<mAP_{1}$ then
train at $LR_{1}=d^{2}\times LR_{0}$ for $N_{1}+1000$
if $mAP_{1}<mAP_{2}$ then
select $w_{2}$
else then
select $w_{1}$
else then
select $w_{0}$
else then
train at $LR_{1}=d^{2}\times LR_{0}$ for $N_{o}+1000$
if $mAP_{0}<mAP_{1}$ then
select $w_{1}$
else then
select $w_{0}$
